# Comorbidities in Children and Adolescents with AIDS Acquired by HIV Vertical Transmission in Vitória, Brazil

**DOI:** 10.1371/journal.pone.0082027

**Published:** 2013-12-04

**Authors:** Sandra F. Moreira-Silva, Eliana Zandonade, Diana O. Frauches, Elisa A. Machado, Lays Ignacia A. Lopes, Lívia L. Duque, Polyana P. Querido, Angélica E. Miranda

**Affiliations:** 1 Infectious Diseases Ward, Nossa Senhora da Glória State Children’s Hospital (Serviço de Infectologia do Hospital Estadual Infantil Nossa Senhora da Glória – SI-HEINSG), Vitória-Espírito Santo (ES), Brazil; 2 Department of Statistics, Federal University of Espírito Santo (Universidade Federal do Espírito Santo – UFES), Vitória-Espírito Santo (ES), Brazil; 3 Post-Graduate Program in Infectious Diseases, Federal University of Espírito Santo (Universidade Federal do Espírito Santo – UFES). Vitória-Espírito Santo (ES), Brazil; University of Pittsburgh, United States of America

## Abstract

**Background:**

Studying diseases associated with AIDS is essential for establishing intervention strategies because comorbidities can lead to death. The objectives were to describe the frequency of comorbidities and verify their distribution according to demographic, epidemiological and clinical data as well as to classify diseases in children and adolescents with AIDS in Vitória, Brazil.

**Methods:**

A retrospective cohort study was conducted among children with AIDS, as defined according to the criteria established by the Ministry of Health, who acquired HIV via vertical transmission, were aged 0 to 18 years, and were monitored at a referral hospital from January 2001 to December 2011.

**Results:**

A total of 177 patients were included, of whom 97 were female (55%). There were 60 patients (34%) <1 year old, 67 patients (38%) between the ages of 1 and 5, and 50 patients (28%) ≥6 years of age included at the time of admission to the Infectious Diseases Ward. Regarding clinical-immunological classification, 146 patients (82.5%) showed moderate/severe forms of the disease at the time of admission into the Ward, and 26 patients (14.7%) died during the study. The most common clinical signs were hepatomegaly (81.62%), splenomegaly (63.8%), lymphadenopathy (68.4%) and persistent fever (32.8%). The most common comorbidities were anaemia (67.2%), pneumonia/septicaemia/acute bacterial meningitis (ABM) (64.2%), acute otitis media (AOM)/recurrent sinusitis (55.4%), recurrent severe bacterial infections (47.4%) and dermatitis (43.1%). An association between severe clinical-immunological classification and admission to the Ward for children aged less than one year old was found for several comorbidities (p<0.001).

**Conclusion:**

Delayed diagnosis was observed because the majority of patients were admitted to the Infectious Diseases Ward at ≥1 year of age and were already presenting with serious diseases. The general paediatrician should be alert to this possibility to make an early diagnosis in children infected with HIV.

## Introduction

The use of antiretroviral therapy (ART) has reduced the morbidity and mortality of AIDS cases and has altered the pattern of the causes of death in these patients. Therefore, studying the diseases associated with AIDS is an important tool for establishing intervention strategies for this population [1]. 

The clinical course of AIDS is faster in children than in adults due to their immunological immaturity [2]. Diagnosis in children is a challenge because clinical presentations resemble other common childhood diseases. Clinical manifestations include the following: prolonged or recurrent fever (often considered to be of unknown origin); recurrent or chronic diarrhoea; generalised lymphadenopathy; persistent or chronic cough; recurrent upper respiratory tract infections (URTI), including sinusitis and otitis; recurrent pneumonia; persistent oral candidiasis; delayed somatic growth; skin lesions, especially eczema; hepatosplenomegaly; and delayed neuropsychomotor development [3].

Early diagnosis of HIV infection, both in children born to seropositive mothers before or during pregnancy or childbirth and in children with nonspecific constitutional symptoms who require recurrent medical attention, will determine the prognosis of these children [4]. Children diagnosed as infected with HIV have a higher frequency of infections, and the infections are often more severe. Follow-up of these children is important to educate the families about the seriousness of AIDS and its consequences for quality of life [5].

In the state of Espírito Santo (Brazil) between 1985 and December of 2011, there were 8,505 reported cases of AIDS, and 377 of these cases were in children under the age of 13 years, comprising 4.4% of all cases. Almost all of these cases occurred through vertical transmission. There has been a considerable decrease in this type of exposure in the state of Espírito Santo, as shown by the number of cases observed in children under 5 years of age, which fell from 27 cases in 2000 to 3 cases in 2011. The incidence rate of AIDS in children less than 5 years old in 2011 was 2.49/100.000inhabitant. However, new cases still occur each year, and primary care for expectant mothers should be complete and of better quality [6].

The goal of this study was to describe the frequency of comorbidities and to verify their distributions according to demographic, epidemiological and clinical data for the classification of cases of children and adolescents with AIDS in Vitória, Espírito Santo, Brazil.

## Methods

This study was submitted and approved by the Research Ethics Committee of the “Nossa Senhora da Glória” State Children’s Hospital (Hospital Estadual Infantil Nossa Senhora da Glória – HEINSG) under number 50/2009. Children’s parents or legal tutors signed the written informed consent.

A retrospective cohort study was conducted in children infected with HIV through vertical transmission who were observed at a paediatric AIDS referral hospital between January 2001 and December 2011. This hospital is linked to the Unified Health System (Sistema Único de Saúde – SUS) of the state of Espírito Santo, and it is a tertiary referral hospital for urgent care/emergencies and paediatric specialties for populations 0-18 years of age.

The population of this study consisted of all patients with AIDS acquired by HIV vertical transmission, aged 0 to 18 years, with a diagnosis defined according to the criteria established by the Brazilian Ministry of Health [4].

The study protocol considered the follow-up period for patients in the Infectious Diseases Ward and included the collection of demographic information, data related to family and diagnostic condition, plasma HIV viral load, CD4+ T lymphocyte count, congenital syphilis, and comorbidities according to the criteria established by the Centers for Disease Control and Prevention (CDC), in accordance with the period in which they were diagnosed [7]. All of the infections were classified with presumptive or definitive diagnoses according to the criteria used by the Brazilian Ministry of Health to define paediatric AIDS [4]. Suggestive diagnoses were accepted in cases of pulmonary tuberculosis and pneumonia caused by *P. jirovecii* due to diagnostic difficulty. The invasive bacterial infections considered were pneumonia, septicaemia and meningitis.

The data were processed using the Statistical Package for the Social Sciences (SPSS) for Windows, version 18.0. To evaluate the information about plasma HIV viral load and CD4+ T lymphocyte counts, the means, medians and interquartile ranges (IQRs) were computed. All of the other variables were analysed by calculating the relative frequency. A chi-squared test or Fisher’s exact test, whichever was the most appropriate, was used to measure the association between comorbidities (outcome) and the severity of the clinical-immunological changes as well as age at time of admission to the Infectious Diseases Ward. The level of significance considered was 5%.

## Results

**Figure 1:**
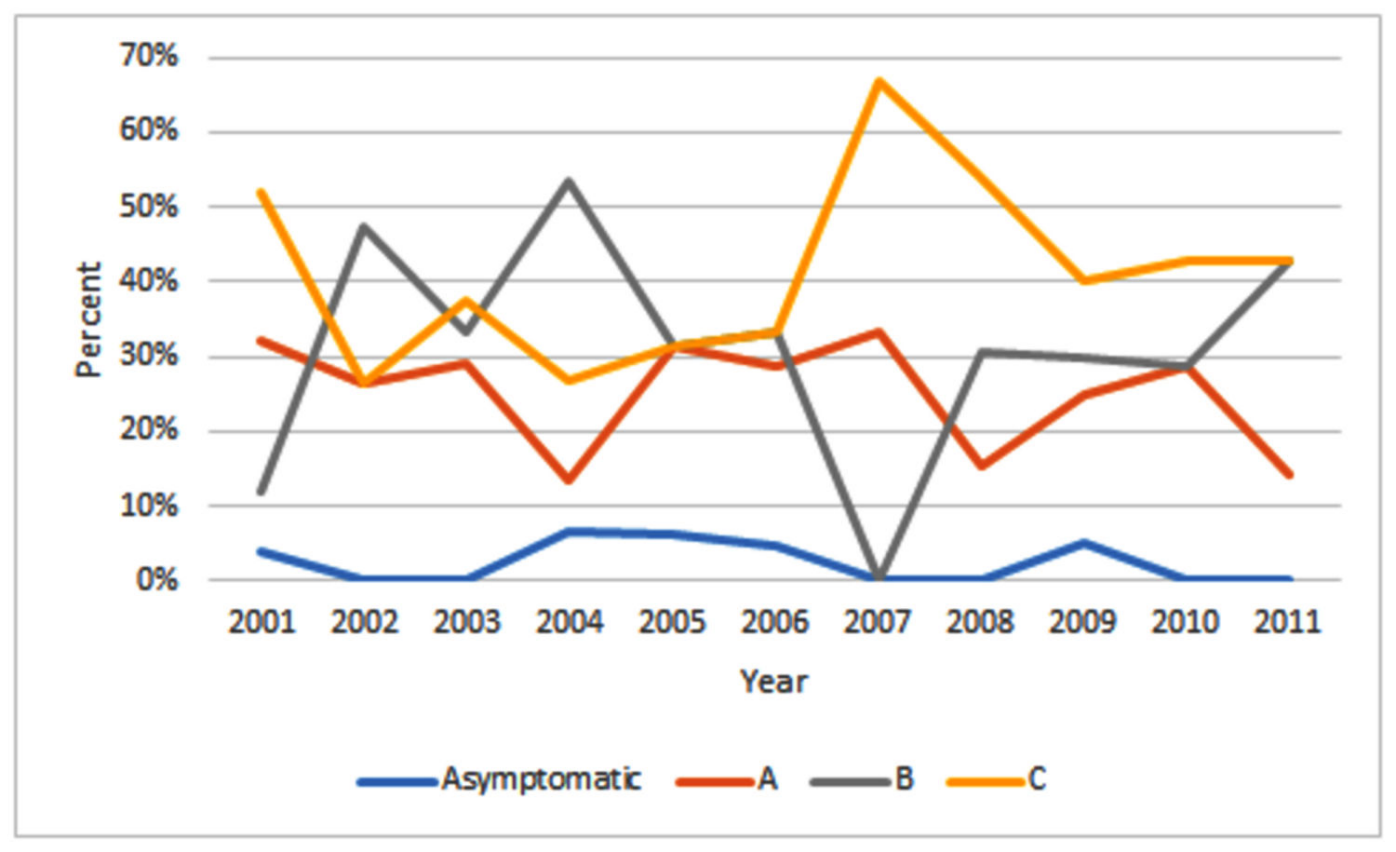
Temporal trend analysis during the 11 years of cases of AIDS in children in Vitoria, Brazil, regarding CDC pediatric HIV disease categories (A, B and C).

This study included 177 patients with AIDS due to HIV vertical transmission; 80 patients (45%) were male, and 97 patients (54.8%) were female. There was a predominance of mixed race (111; 62.7%), and their ages were distributed as follows: 33.9% (60) less than 1 year old, 37.9% (67) between 1 and 5 years old, and 28.2% (50) 6 or more years old. The majority of patients, 124 (70%), came from five municipalities that make up the metropolitan area of Vitória. A total of 106 patients (106/175, 60.6%) lived with their parents. There was no difference rregarding temporal trend analysis of cases of AIDS in children regarding CDC pediatric HIV disease categories (Figure **1**). 

The median patient follow-up was 5 years, with an interquartile range of 2 to 8 years and a maximum follow-up of 11 years. At the time of admission to the Infectious Diseases Ward, 146 patients (82.5%) showed a moderate/severe clinical-immunological classification (B2, B3, C1, C2, or C3). Diagnosis was made in 75/174 children (43%) due to illness. During this period, there were 26 deaths (14.7%), but two cases were not included in the study, one death occurred after a hospital transfer and one death after treatment abandonment (Table **1**). A total of 11 patients (6.2%) were transferred, 8 patients (4.5%) did not continue follow-up and 158 patients (89.3%) continued follow-up, including the 24 deaths occurred during this period.

**Table 1:** Profile of patients with AIDS due to vertical HIV transmission observed at SI-HEINSG in the period of 2001 to 2011, Vitória - Brazil (N=177).

**Variable**	**Categories**	**N**	**%**
Sex	Male	80	45.0
	Female	97	55.0
Race/colour	Caucasian	34	19.0
	Black	30	17.0
	Mixed Race	111	63.0
	Asian	2	1.0
Age at admission to the	Less than 1 year	60	34.0
Infectious Diseases Ward	1 to 5 years	67	38.0
	6 years or more	50	28.0
Area of residence	Urban	124	70.0
	Rural	44	24.9
	Other states	9	5.1
Caregiver	Father/mother	106	60.6
	Family members	44	25.1
	Adoptive	11	6.3
	Institution	14	8.0
Case diagnosis (N=174)	Due to child illness	75	43.0
HIV viral load	< 100,000 copies/ml	72 41.7	
	≥ 100,000 copies/ml	102 58.3	
Nadir CD4 < 15%	Yes	85	48.5
	No	92	52.5
Current situation	Alive	151	85.3
	Deceased	26	14.7
Clinical-immunological category	A1	31	17.5
	A2	17	9.6
	A3	2	1.1
	B1	24	13.6
	B2	20	11.3
	B3	11	6.2
	C1	15	8.5
	C2	15	8.5
	C3	42	23.7

Among the 175 patients with available data on their first CD4+ T lymphocyte counts, the median was 845.0 cells/µL (IQR: 440-1352). The median viral load was 18,000 copies/mL (IQR: 30,900-750,000), and the log median initial HIV viral load was 5.25 (IQR: 4.47 - 5.98). Among the 162 patients with available data on their first CD8+ T lymphocyte counts, the median was 1,270.0 (IQR: 839.0 - 2,091.8). 

The main clinical signs shown by patients were hepatomegaly in 81.6% (142/174), splenomegaly in 63.8% (111/174), lymphadenopathy in 68.4% (119/174) and persistent fever in 32.8% (57/174). Regarding the frequency of diagnosed comorbidities, it was observed that chronic anaemia (117/174, 67.2%), a first episode of invasive bacterial infection (pneumonia [PN], acute bacterial meningitis [ABM] or septicaemia [111/173, 64.2%]), acute otitis media (AOM) or recurrent sinusitis (97/175, 55.4%), other episodes of severe bacterial infections (PN, ABM or septicaemia, 82/173, 7.4%), dermatitis (75/174, 43.1%), prolonged diarrhoea or gastroenteritis (GE) (71/174, 40.8%) and candidiasis (60/174, 34.5%) were the most frequent (in decreasing order) and that mycobacterial disease, progressive multifocal leukoencephalopathy and cryptosporidiosis were the least frequent; furthermore, there were no cases of pulmonary herpes simplex or histoplasmosis. There were 24/177 cases (13.6%) of tuberculosis, and the state of immunosuppression in these cases was significant (p=0.001). None of the patients with tuberculosis made use of isoniazid prophylaxis, and only 3 patients with TB/HIV co-infection showed a PPD ≥ 5 mm (Table **2**).

**Table 2:** Diagnosed comorbidities in patients with AIDS due to HIV vertical transmission treated at SI-HEINSG in the period of 2001 to 2011, Vitória/ES - Brazil (N=177).

**Diagnosed comorbidities**	**N**	**%**
Chronic anaemia	117	67.2
First episode of PN, ABM or septicaemia	111	64.2
Acute otitis media or recurrent sinusitis	97	55.4
Recurrent severe bacterial infection	82	47.4
Dermatitis	75	43.1
Prolonged diarrhoea or gastroenteritis	71	40.8
Candidiasis	60	34.5
Varicella or herpes zoster	50	28.7
Wasting syndrome	39	22.4
*Pneumocystis*	34	19.7
Lymphocytic interstitial pneumonia	34	19.2
HIV-related hepatitis	32	18.4
HIV-related encephalopathy	25	14.4
Thrombocytopaenia	24	13.8
Tuberculosis	24	13.6
Chronic swelling of the parotid gland	23	13.4
Cytomegalovirus infection	17	9.7
Dyslipidaemia	12	6.9
Toxoplasmosis	12	6.9
Pancreatitis	9	5.2
Cardiomyopathy	5	2.9
Congenital syphilis	5	2.8
Gingivostomatitis or recurrent herpes	5	2.9

Others: renal disorders, cryptococcosis, isosporiasis, lymphoma, cryptosporidiosis, progressive multifocal leukoencephalopathy and disseminated mycobacteriosis.

PN: pneumonia; ABM: acute bacterial meningitis.

The comorbidities that showed a significant difference (p<0.05) in relation to the clinical-immunological classification were as follows: chronic anaemia; a first episode of PN, ABM or septicaemia; prolonged diarrhoea or GE; other episodes of serious bacterial infections; tuberculosis; *Pneumocystis jirovecii* pneumonia; wasting syndrome; HIV-related encephalopathy; HIV-related hepatitis; thrombocytopenia; candidiasis; and dermatitis (Table **3**).

**Table 3:** Relationship between comorbidity and clinical-immunological abnormalities in patients with AIDS due to HIV vertical transmission treated at SI-HEINSG in the period of 2001 to 2011 (N=177).

**Comorbidity / Clinical-immunological abnormalities**	Severe*		**Not severe**		**p-value**
	**N**	**%**	**N**	**%**	
Chronic anaemia	67	79.8	50	55.6	**0.001**
Chronic swelling of the parotid gland	7	8.5	16	17.8	0.059
Dermatitis	42	50.6	33	36.3	**0.040**
Acute otitis media or recurrent sinusitis	45	53.6	52	57.1	0.373
Prolonged diarrhoea or gastroenteritis	49	58.3	22	24.4	**0.000**
HIV-related hepatitis	21	25.3	11	12.1	**0.020**
Thrombocytopaenia	17	20.2	7	7.8	**0.015**
Varicella or herpes zoster	23	27.7	27	29.7	0.454
First episode of PN, ABM or septicaemia	74	89.2	37	41.1	**0.000**
Lymphocytic interstitial pneumonia	20	23.8	14	15.6	0.119
Tuberculosis	19	22.4	5	5.4	**0.001**
HIV-related encephalopathy	21	25.0	4	4.4	**0.000**
Recurrent severe bacterial infection	67	79.8	15	16.9	**0.000**
*Pneumocystis*	31	37.3	3	3.3	**0.000**
Pancreatitis	8	9.5	1	1.1	**0.013**
Dyslipidaemia	7	8.4	5	5.6	0.328
Candidiasis	49	59.8	11	12.2	**0.000**
Cytomegalovirus infection	12	14.1	5	5.5	**0.046**
Toxoplasmosis	5	5.9	7	7.8	0.424

* Classification: B3-C1, 2, 3

Regarding the patients’ age at the time of admission to the Infectious Diseases Ward (Table **4**), children less than one year old showed a difference in the frequency of several diseases to a statistically significant degree: anaemia; a first episode of PN, ABM or septicaemia; prolonged diarrhoea or GE; HIV-related hepatitis; candidiasis; others episodes of severe bacterial infections; *Pneumocystis jirovecii* pneumonia; wasting syndrome; and dermatitis. Chronic swelling of the parotid gland, lymphadenopathy, and lymphocytic interstitial pneumonia (LIP) were more frequent in children older than one year.

**Table 4:** Relationship between comorbidity and age range at time of admission to the service for patients with AIDS due to HIV vertical transmission at SI-HEINSG in the period of 2001 to 2011 (N=177).

**Comorbidity/ Age of admission to service**	**Less than 1**		**1 to 5 years**		**6 or more**		**p-value**
	**N**	**%**	**N**	**%**	**N**	**%**	
Chronic swelling of the parotid gland	1	1.7	15	22.7	7	14.6	**0.003**
Dermatitis	29	49.2	28	41.8	18	37.5	0.463
Acute otitis media or recurrent sinusitis	29	49.2	35	52.2	33	67.3	0.133
Chronic anaemia	49	83.1	44	65.7	24	50.0	**0.010**
Prolonged diarrhoea or gastroenteritis	34	57.6	20	30.3	17	34.7	**0.005**
HIV-related hepatitis	19	32.7	10	14.9	3	6.1	**0.001**
Varicella or herpes zoster	13	22.4	24	35.8	13	26.5	0.236
First episode of PN, ABM or septicaemia	44	74.6	43	65.2	24	50.0	**0.030**
Thrombocytopaenia	9	15.2	12	17.9	3	6.3	0.187
Cardiomyopathy	1	1.7	2	3.0	2	4.3	a
Renal disorder	0	0.0	1	1.5	2	4.3	a
Lymphocytic interstitial pneumonia	6	10.2	17	25.4	11	23.9	0.078
Tuberculosis	13	21.7	6	8.9	5	10.0	0.077
Cryptococcosis	2	3.4	0	0.0	1	2.1	a
Cryptosporidiosis	0	0.0	1	1.5	0	0.0	a
HIV-related encephalopathy	11	18.6	11	16.4	3	6.3	0.159
Histoplasmosis	0	0.0	0	0.0	0	0.0	a
Recurrent severe bacterial infection	37	62.7	28	41.8	17	36.2	**0.012**
Isosporiasis	1	16.7	1	1.5	0	0.0	a
Leukoencephalopathy	0	0.0	0	0.0	1	2.1	a
Lymphoma	0	0.0	1	1.5	1	2.1	a
*Pneumocystis*	20	33.9	11	16.7	3	6.3	**0.001**
Mycobacteriosis	0	0.0	1	1.5	0	0.0	a
Wasting syndrome	17	28.8	11	16.4	11	22.9	0.249
Pancreatitis	2	3.4	2	3.0	5	10.4	a
Dyslipidaemia	2	3.4	6	8.9	4	8.5	a
Candidiasis	30	50.8	16	24.2	12	24.5	**0.002**
Cytomegalovirus infection	6	10.0	8	11.9	3	61.2	0.574
Toxoplasmosis	3	5.0	3	4.5	6	12.5	a
Congenital syphilis	4	6.8	1	1.5	0	0.0	a

a: It was not possible to calculate the hypothesis test because some of the cells were equal to zero.

Table **5** describes the characteristics of mothers of the 177 children. The type of HIV exposure was sexual transmission in 134 mothers (75.7%), the use of injectable drugs in 16 mothers (9%), blood transfusion in 2 mothers (1.1%) and unknown in 25 mothers (14.2%). In 118 cases (66.7%), maternal HIV was diagnosed after the delivery of the child, and only 12/175 mothers (6.9%) underwent the complete protocol to prevent HIV vertical transmission (gestation/delivery/NB and no breastfeeding). A total of 56/89 mothers (62.9%) breastfed their infants. Regarding antenatal care, 95 mothers (53.7%) had access to antenatal care, but of those, 49 women (51.6%) were diagnosed with HIV after delivery. Delivery was vaginal in 52/86 (60.5%) of these women.

**Table 5:** Characteristics of the mothers of patients with AIDS due to HIV vertical transmission observed at SI-HEINSG in the period of 2001 to 2011 in Vitória/ES -Brazil (N=177).

**Variable**	**Categories**	**N**	**%**
Category of AIDS exposure of the mother	Sexual	134	75.7
	Use of injectable drugs	16	9
	Transfusion	2	1.1
	Unknown	25	14.2
Mother received prenatal care	Yes	95	53.7
	No	77	43.5
	Unknown	5	2.8
Mother’s HIV diagnosis	Prior to delivery	47	26.5
	At delivery	9	5.1
	After delivery	118	66.7
	Unknown	3	1.7
Brazilian national PMTCT*	Completed	12	6.9
protocol (N=175)	Incomplete or not performed	163	93.1
Breastfeeding (N=155)	Yes	113	72.9
	No	42	27.1
Type of delivery	Vaginal	105	59.2
	Caesarean	40	22.6
	Unknown	32	18.1
Age of the mother	27 years (±5.89)	Min 15 Max 44	-

* PMTCT: Prevention Mother to Child Transmission

## Discussion

In this study, a high frequency of comorbidities was observed in children treated at a tertiary referral hospital for urgent care/emergencies and paediatric specialties. This high frequency can be explained by this hospital being the main public paediatric referral hospital for AIDS in the state of Espírito Santo, and many children come in at advanced ages with a late diagnosis of HIV exposure/infection. Children infected with HIV by vertical transmission, according to a recent study, exhibit a bimodal clinical progression. Thus, 10-30% of patients, called rapid progressors, begin to show symptoms of AIDS in their first year of life, and a small group of patients, called slow progressors, remain asymptomatic for many years and can even reach school age unaffected [8].

Several children in our study showed moderate and severe manifestations, with 23.7% patients classified as clinical category C3, most of whom had high HIV viral loads. In a meta-analysis of mortality predictors among untreated HIV-infected children in Africa and Brazil, the CD4% and CD4+ T lymphocyte count were the most important predictors of mortality, followed by low body weight for age and haemoglobin level [9]. Other Brazilian studies described clinical symptoms and immunological disorders in children and adolescents infected with HIV and the disease progression [10,11,12,13].

Anaemia was the most prevalent comorbidity in all age groups at the time of admission to the hospital service, and it was more frequent in children less than 1 year old and in severe clinical-immunological category C3 patients. Anaemia was more prevalent in our study and had a statistically significant (p=0.007) association in children with severe clinical-immunological alterations. Recent studies in HIV-positive individuals have shown that anaemia is one of the most common haematological manifestations, some data have suggested that bone marrow suppression is the primary pathophysiological mechanism of anaemia in these patients [14,15]. Bacterial infections were frequent in our sample, more than sixty percent of the children develop at least one episode of bacteraemia, even with ART use, most likely due to a late diagnosis of HIV infection. An American study found a significant decrease in the incidence of bacteraemia and a prolongation of the time to the first episode of bacteraemia after the advent of potent ART [16].

A total of 14% children in our sample presented with tuberculosis (TB). The severe state of immunosuppression in these cases was significant (p=0.001), and tuberculosis was significantly associated with an age at admission to the Infectious Diseases Ward of less than one year (p=0.02). A previous study on the prevalence of AIDS in children diagnosed with active TB in the state of Espírito Santo showed 411 cases of paediatric TB from 2000 to 2006, and 27 of these children (7%) were co-infected with AIDS [17]. In a Chilean study, of 246 children with AIDS due to HIV vertical transmission, six presented with TB/HIV co-infection [18].

Another relatively common manifestation in children with AIDS that is rarely observed in adults is lymphocytic interstitial pneumonia (LIP), which is characterised by diffuse chronic interstitial pneumonia with a follicular lymphocytic infiltrate that may progress to cor pulmonale due to cardiac abnormalities, resulting in a continuous increase in pulmonary vascular resistance [19]. The frequency of LIP in our study was 19.5%, and the LIP was significantly associated with the group of children over one year of age (p=0.001). *Pneumocystis jirovecii* pneumonia has the highest incidence in HIV-infected children in their first year of life, with most cases occurring between 3 and 6 months of age [1,20].

Reducing vertical HIV transmission becomes viable when infected pregnant women are identified and treated with antiretroviral drugs during pregnancy and delivery, and the newborn infant also is treated at birth [21]. Although Brazilian Government offers free antiretroviral therapy, the proportion of HIV-positive pregnant women who do not have access to the prophylactic measures recommended by the Brazilian national PMTCT protocol, and who ultimately fail to be tested for HIV, remains high, either as a function of the women’s social condition or due to inadequacies in the health-care system. Late detection of HIV infection during antenatal care represents an opportunity to intervene in the case of the HIV-infected patient that is lost, thus limiting the possibilities of reducing the number of pediatric cases caused by vertical transmission [22].

Although retrospective data are not ideal for assessing the occurrence of comorbidities and the association of epidemiological and clinical data, their application is justified because implementing health care assistance for these mothers and children is important to demonstrate the susceptibility of the population to this form of HIV transmission. The possibility of response bias by the mothers cannot be discarded because there is a tendency to give more socially acceptable answers. The lack of accuracy regarding data on monitoring during pregnancy and at childbirth also cannot be excluded. 

Despite significant reductions in recent years due to ART, opportunistic diseases are still common in Brazilian children with AIDS, especially bacterial diseases. These data reinforce the need to intensify efforts in preventing vertical transmission, early diagnosis of infection, and better paediatric care. Therefore, paediatricians should always be suspicious of and consider the possibility of an HIV/AIDS diagnosis to make an early diagnosis in HIV-infected children.
